# The complete mitochondrial and plastid genomes of the invasive marine red alga *Caulacanthus okamurae* (Caulacanthaceae, Rhodophyta) from Moss Landing, California, USA

**DOI:** 10.1080/23802359.2020.1763870

**Published:** 2020-05-13

**Authors:** Hartnell College Genomics Group, Alejandro Aguilar, Tania J. Ahumada, Noemi Amezcua Moreno, Jacob Bohn, Danilo E. Bustamante, Martha S. Calderon, Edgar Cardoso, Rocio Carranza, Maria Castillo, Elizabeth Cazares, Emmanuel Cazares, Janelle K. Companion, Jeanette Cruz, Neovid Cuevas, Lizbeth De La Torre, Daniel P. Dietz, Kathleen M. Fernando, Bryan Garcia, Pamela Gomez, Bianca Gonzales-Miramontes, Yuviana Hernandez, Kayla Huaracha, Jeffery R. Hughey, Gabriela Lazaro, Feifei Zhai Lorenzo, Diana Medrano, Ailen Mendoza, Daniel Mendoza, Adam Mohssin, Jeanette Orozco Medina, Aislinn Pacheco, Gustavo Palacios Ruvalcaba, Jankiben Patel, Jatin Patel, Samantha Patino, Karina Perez-Alfaro, Alejandra N. Ponce, Jasmine G. Poso, Gabriel Ramirez, Hector A. Ramirez, Nathalie Resendiz, Rachelle Reyno, Diego Rodriguez, Ian A. Russell, Paola Saenz-Verdugo, Angel S. Carmona, Fabian Sanchez, Sabrina X. Sheffer, Carmen Solorio, Alejandra Soto Trujillo, Geraldine S. Vasaya, Victoria Velasquez Lopez

**Affiliations:** a Division of Mathematics, Science, and Engineering, Hartnell College, Salinas, CA, USA; b Instituto de Investigación para el Desarrollo Sustentable de Ceja de Selva (INDES-CES), Universidad Nacional Toribio Rodríguez de Mendoza, Amazonas, Peru; c Laboratorio de Ecosistemas Marinos Antárticos y Sub-antárticos (LEMAS), Universidad de Magallanes, Punta Arenas, Chile

**Keywords:** *Caulacanthus*, *Caulacanthus ustulatus*, invasive species, mitogenome, plastid genome

## Abstract

*Caulacanthus okamurae* is an invasive red alga that forms extensive mats in sheltered marine habitats around the world. To determine its genomic structure and genetic relationship to native and other non-native populations of *C. okamurae*, high-throughput sequencing analysis was performed on an introduced specimen from Bennett Slough, Moss Landing, California, USA. Assembly of 23,146,595 filtered 150 bp paired-end Illumina sequencing reads yielded its complete mitogenome (GenBank accession MT193839) and plastid genome (GenBank accession MT193838). The mitogenome is 25,995 bp in length and contains 50 genes. The plastid genome is 173,516 bp and contains 234 genes. Comparison of the organellar chromosomes to other Gigartinales revealed a high-level of gene synteny. BLAST analysis of marker sequences (*rbc*L, *cox*1, *cox2*) of *C. okamurae* from Moss Landing identified four identical DNA sequences: one from a specimen from a native population of *C. okamurae* from South Korea and three from specimens representing invasive populations from France, Spain, and the USA. These genetic results confirm the presence of *C. okamurae* in central California, USA, and represent the first complete mitogenome and plastid genome from the Caulacanthaceae.

*Caulacanthus okamurae* Yamada (1933), Japanese name Isodantû, is a densely tufted, diminutive, dark yellow to purple brown intertidal red seaweed, originally named from specimens collected from the Ryukyu Islands, Japan. The species is native to China, Japan, South Korea, and Taiwan, but is invasive in other parts of the world (Fofonoff et al. 2020). *Caulacanthus okamurae* was long regarded as a synonym of *C. ustulatus* (Mertens ex Turner) Kützing (West and Calumpong 1990; Zuccarello et al. 2002), but is now considered a distinct species. In Bennett and Elkhorn Sloughs, Moss Landing, California, an introduced population of *C. okamurae* forms extensive carpet-like mats that cover the substrate. Here, we performed whole genome sequencing on a specimen of *C. okamurae* from Bennett Slough, Moss Landing (36°49′01.3ʺN, 121°47′16.3ʺW) to determine its mitochondrial and plastid chromosomal content and structure, as well as confirm its identity and relationship to other invasive populations of *C. okamurae* from around the world.

DNA was extracted from *C. okamurae* (Specimen Voucher – UC 2085024) following the protocol of Lindstrom et al. (2011). The 150 bp PE Illumina library construction and sequencing was performed by myGenomics, LLC (Alpharetta, Georgia, USA). The genomes were assembled using default *de novo* settings in MEGAHIT (Li et al. 2015) and annotated using Geneious Prime (Biomatters Limited, Auckland, New Zealand), NCBI ORFfinder, and tRNAscan-SE 1.21 (Schattner et al. 2005). The *C. okamurae* mitogenome was aligned to other mitogenomes with MAFFT (Katoh and Standley 2013). The phylogenetic analysis was executed at Trex-online (Boc et al. 2012) with the GTR + gamma model and 1000 bootstraps. The tree was visualized with TreeDyn 198.3 at Phylogeny.fr (Dereeper et al. 2008).

The mitogenome of *C. okamurae* is 25,995 bp in length and contains 50 genes. It is A + T rich (69.3%) and includes 23 tRNA, 5 ribosomal proteins, 4 ATP synthase, 2 rRNA, and 16 other genes involved in electron transport and oxidative phosphorylation. The mitogenome of *C. okamurae* differed from other Gigartinales in tRNA tract composition. The plastid genome of *C. okamurae* is 173,516 bp and contains 233 genes. The genome is also A + T biased (69.9%) and contains 47 ribosomal proteins, 29 tRNA, 30 photosystem I and II, 32 ycf, 10 phycobiliprotein, 9 cytochrome b/f complex, 8 ATP synthase, 4 RNA polymerase, 4 orfs, 3 rRNA, and 57 other genes. The plastid genome was highly similar in length, content, and organization to other Gigartinales (Janouškovec et al. 2013; Yang et al. 2015; Sissini et al. 2016).

Phylogenetic analysis of the *C. okamurae* mitogenome places it in an unresolved branch sister to *Chondrus crispus* Stackhouse and *Mastocarpus papillatus* (C. Agardh) Kützing (Figure 1). A BLAST search of *cox*1, *cox2*, and *rbc*L sequences of *C. okamurae* from Moss Landing identified a single DNA match to a native specimen from Pusan, South Korea, and three matching sequences representing introduced populations of *C. okamurae* from Roscoff, France, Spain, and Washington, USA. *Caulacanthus okamurae* differs significantly in pairwise genetic distance from *C. ustulatus* (Mertens ex Turner) Kützing (Type Locality: Cádiz, Spain), to which it was previously confused, by 14.6% for *cox1*, 14.4–20.4% for *cox2*, and 6.8–9.1% for *rbc*L. These genetic analyses confirm the presence of *C. okamurae* in Moss Landing, California, and contribute to the genomics and systematics of this highly invasive species.

**Figure 1.**
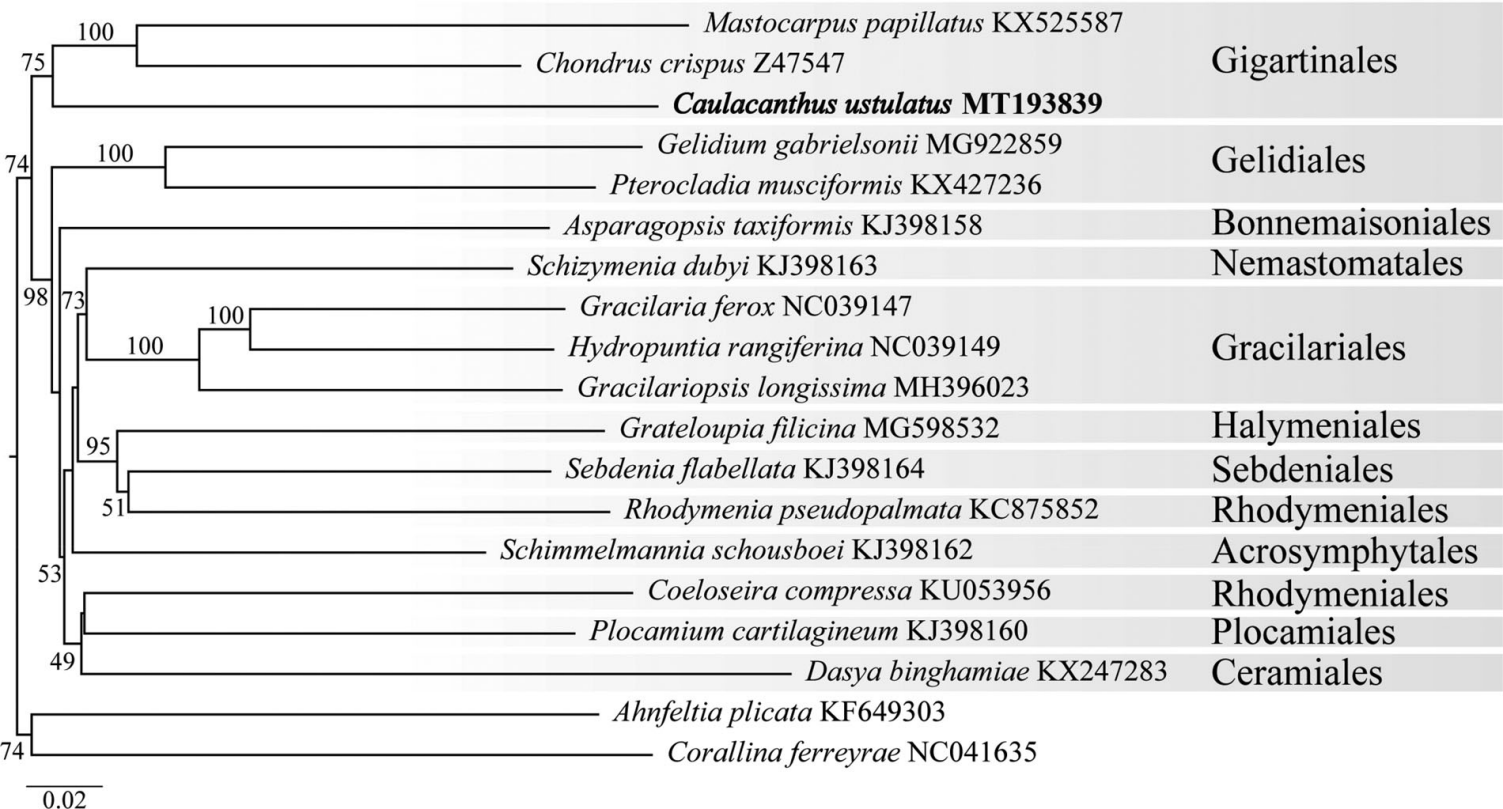
Maximum likelihood phylogram of the complete mitogenome of *Caulacanthus okamurae* (GenBank Accession MT193839) and related red algae. Numbers along branches are RaxML bootstrap supports based on 1000 replicates. The legend below represents the scale for nucleotide substitutions.

## Data Availability

The data that support the findings of this study are openly available in GenBank at https://www.ncbi.nlm.nih.gov/nucleotide/, reference numbers MT193838 and MT193839.
